# Complete genome sequences of *Plantago lanceolata* latent virus isolated from asymptomatic ribwort plantain plants in France, Italy, and Spain

**DOI:** 10.1128/mra.00654-25

**Published:** 2025-09-26

**Authors:** Denis Filloux, Serge Galzi, Philippe Roumagnac

**Affiliations:** 1 CIRAD, UMR PHIM 27050, Montpellier, France; 2 PHIM Plant Health Institute, Université de Montpellier, CIRAD, INRAE, Institut Agro, IRD 27037, Montpellier, France; Portland State University, Portland, Oregon, USA

**Keywords:** geminivirus, ribwort plantain

## Abstract

Plantago lanceolata latent virus (PlLV) that infects ribwort plantain, initially discovered in Finland, is also present in Western Europe. Four complete genome sequences of PlLV from France, Italy, and Spain were obtained that shared 94.17%–97.85% genome-wide pairwise identity with complete genomes of PlLV previously obtained from Finland.

## ANNOUNCEMENT

**Fig 1:**
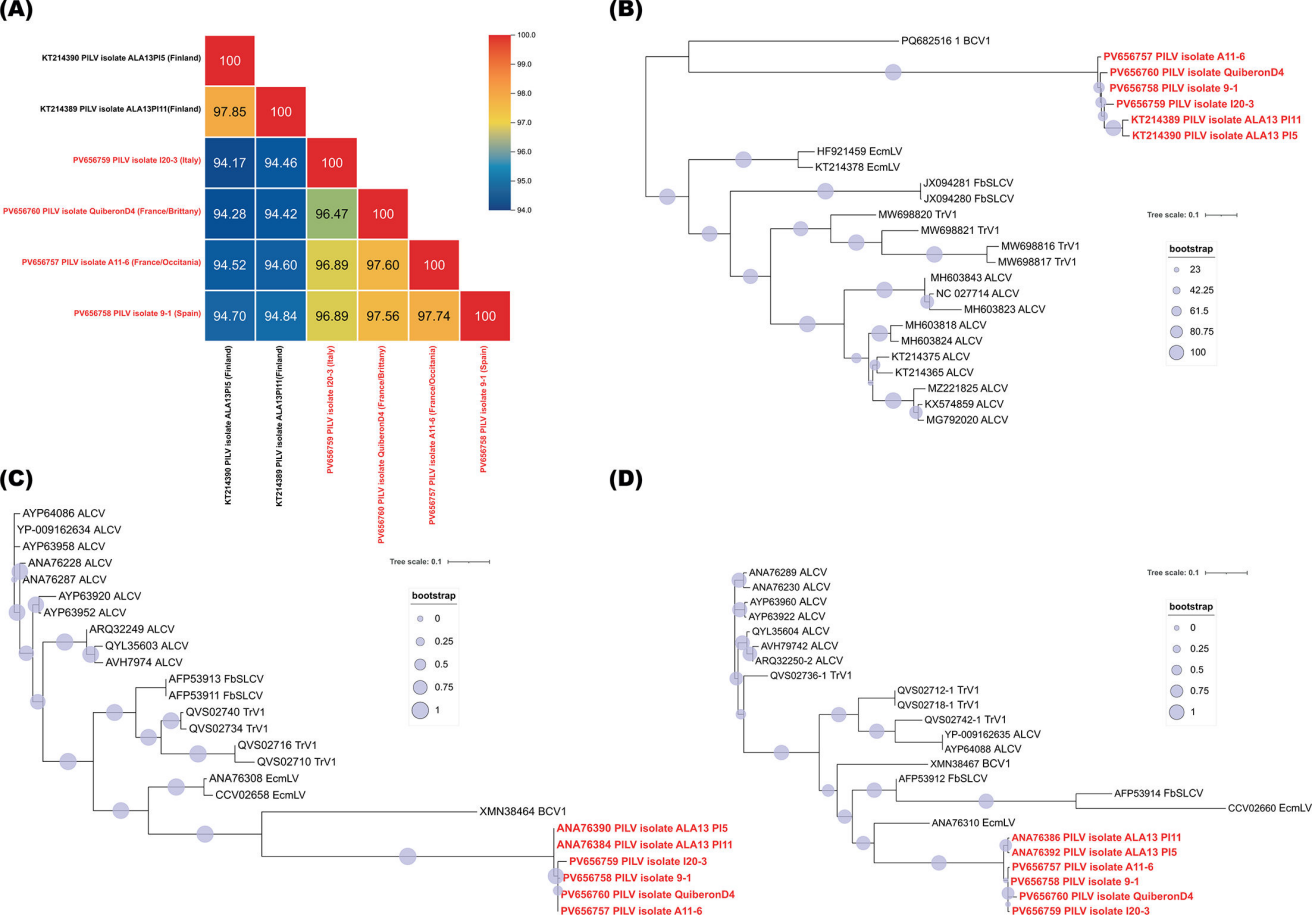
(**A**) Pairwise identity matrix of the complete genome sequences of PlLV isolates from Finland, France, Italy, and Spain. (**B**) Maximum likelihood phylogenetic trees with 1,000 bootstrap replicates of complete capulavirus genome nucleotide sequences. (**C**) Maximum likelihood phylogenetic trees with 1,000 bootstrap replicates of capulavirus capsid protein (CP) amino acid sequences. (**D**) Maximum likelihood phylogenetic trees with 1,000 bootstrap replicates of capulavirus replication-associated protein (REP) amino acid sequences.

Plantago lanceolata latent virus (PlLV) that belongs to the genus *Capulavirus* (*Geminiviridae* family) infects ribwort plantain (*Plantago lanceolata* L.) (1), a perennial herb that is native from Eurasia and is now widespread all over the world (2). This virus was discovered in 2015 from asymptomatic, uncultivated ribwort plantain plants collected in the Åland archipelago of southwestern Finland (1). We here investigated to what extent the virus was present outside of Finland, particularly in Western Europe. One hundred and thirty-one leaves from asymptomatic ribwort plantain plants were collected in 2018 from France (94 samples), Italy (17 samples), and Spain (20 samples). Total DNA of ribwort plantain plants was extracted using the DNeasy Plant Mini Kit (Qiagen, Germany). PCR-mediated detection of PlLV from the 131 plant samples was performed using PCR primers F-PlLV_729 (5′-AAGGGAAAGGCTGGTTATGG-3′) and R-PlLV_1013 (5′-GAATCTCTTCTCTGAATCGTGGTC-3′). Amplification conditions consisted of 95°C for 5 min; 30 cycles at 95°C for 45 s, 50°C for 45 s, 72°C for 30 s; and 72°C for 5 min. The PCR amplicons corresponding to visible agarose gel bands were Sanger sequenced by Azenta (Germany) using the ABI3730xl sequencer (Applied Biosystems). PCR analysis revealed that PlLV was present in 2/94 samples in France, 1/17 samples in Italy, and 3/20 samples in Spain. PCR amplification of the complete genome of 4/6 positive samples from France, Italy, and Spain (one sample was selected from each region: Brittany, Galicia, Languedoc, and Emilia-Romagna; Table 1) was performed using a pair of abutting primers, Pla_pstIF (5′-CTGCAGATCATTGTATAAATACTGTCCCAAATACG-3′) and Pla_pstIR (5′-CTGCAGTATCTGTGATATTTGTATACAAATTTCTGAC-3′), as previously described (1). Amplicon products of approximately 2.8 Kbp were excised, gel-purified, and cloned into the plasmid pGEM-T Easy and further Sanger sequenced by primer walking (Azenta). Four PlLV complete genome sequences were obtained, ranging in size from 2,832 nt to 2,834 nt (%GC content of 41.8%). Pairwise identity analyses of the full genome nucleotide sequences were further carried out using SDT v1.2 using default settings (3), and indicated that the four PlLV complete genome sequences obtained in this study and both complete genomes obtained previously from Finland (1) shared 94.17–97.85% genome-wide pairwise identity (Fig. 1A). The observed degree of similarity exceeds the species demarcation threshold recommended for the *Capulavirus* genus (4), indicating that the four isolates from France, Italy, and Spain could be classified as PlLV variants. The evolutionary relationships of the six PlLV isolates (four from this study and two from the study conducted in Finland) and representative members of capulaviruses were reconstructed using the complete nucleotide genome sequences, the replication-associated protein (REP), and the coat protein (CP) amino acid sequences. Alignment was carried out using MAFFT (5). Block mapping and gathering with entropy (6) were used, and a Maximum-Likelihood tree was inferred by FastTree with 1,000 bootstrap iterations (7). The three phylogenetic analyses based on the complete nucleotide genome sequences (Fig. 1B), the CP protein (Fig. 1C), and the REP protein (Fig. 1D) sequences all indicate that the four PlLV genomes from France, Italy, and Spain cluster with the other two Finnish isolates previously described. This study demonstrates that PlLV is not confined to a few islands in the Baltic Sea but is widespread in Western Europe.

**TABLE 1:** Metadata of the four PlLV isolates from France, Italy, and Spain

PlLV isolate	Collection date	Location	GPS coordinates	Accession number
A11-6	July 2018	Assas (Languedoc, France)	43.702240 3.8893762	PV656757
9-1	August 2018	Carnota (Galicia, Spain)	42.80580 −9.123135	PV656758
I20-3	May 2018	Baricella (Emilia-Romagna, Italy)	44.644715 11.531441	PV656759
QuiberonD4	July 2018	Saint Pierre Quiberon (Brittany, France)	47.49957 −3.119324	PV656760

## Data Availability

The complete genome sequences of the four PlLV isolates from France, Italy, and Spain reported in this manuscript have been deposited under GenBank accession numbers PV656757, PV656758, PV656759, and PV656760. All Sanger reads have been submitted to the NCBI’s SRA database under GenBank accession numbers PRJNA1277769 (SRX29201394, SRX29201393, SRX29201392 and SRX29201391).
